# Functional Elbow Reconstruction After Complete Ulnar Resection for Ewing′s Sarcoma: Radial Neck Transposition as a Biomechanical Alternative

**DOI:** 10.1155/cro/1607267

**Published:** 2026-01-25

**Authors:** Fernando Brasil do Couto Filho, Deivid Ramos dos Santos, Luiz Claudio Campelo Barbosa, Felipe Guimarães Magno, Henrique Ribeiro Rodrigues Neto, Rodrigo Da Silva Cordeiro, Lorenzo Giordano do Couto, Eurineto Gomes do Nascimento, Raul Martins Barra

**Affiliations:** ^1^ Head of Department of Orthopedic Oncology, Octavio Lobo Pediatric Oncology Hospital, Belém, Pará, Brazil; ^2^ Department of Orthopedic Oncology and State University of Pará-Postgraduate Program in Surgery and Experimental Research, Octavio Lobo Pediatric Oncology Hospital, Belém, Pará, Brazil; ^3^ Department of Orthopedic Oncology, Octavio Lobo Pediatric Oncology Hospital, Belém, Pará, Brazil; ^4^ Department of Orthopedic Oncology, Santa Casa de Misericórdia de São Paulo and Octavio Lobo Pediatric Oncology Hospital, São Paulo, São Paulo, Brazil

**Keywords:** elbow reconstruction, Ewing′s sarcoma, limb salvage, orthopedic oncology, radial neck transposition

## Abstract

**Introduction:**

Ewing′s sarcoma of the ulna is rare, and its wide resection poses challenges for preserving upper limb function. Elbow reconstruction must ensure joint stability and forearm mobility. Conventional alternatives, such as bone grafts and prostheses, have limitations, including a high risk of complications.

**Objective:**

This study was aimed at reporting a case of elbow reconstruction using radial neck transposition to the humeral trochlea after ulnar resection for Ewing′s sarcoma, evaluating the functional outcomes of this technique.

**Methods:**

A 17‐year‐old male patient with Ewing′s sarcoma in the ulnar diaphysis underwent complete ulnar resection. Radial neck transposition to the humeral trochlea was performed. Follow‐up included functional assessment using the Musculoskeletal Tumor Society (MSTS) scale and postoperative imaging.

**Results:**

After 2 years, the patient had an MSTS score of 28/30, with preserved wrist and hand mobility, full pronation, and supination reduced to half. Radiographic follow‐up demonstrated proper alignment of the reconstruction, with no instability or bone resorption.

**Conclusion:**

Radial neck transposition proved to be a viable alternative for elbow reconstruction after ulnar resection, providing stability and functional preservation. Further studies are needed to validate its application on a larger scale.

## 1. Introduction

Chronic pain in the forearm and elbow region is a common complaint in both general and orthopedic clinical practice [1, 2]. In adolescents and young adults, such symptoms are often attributed to overuse syndromes, growth‐related conditions, or minor trauma [3]. However, when pain is persistent, nonresponsive to conservative treatment, and lacking an identifiable mechanical cause, less common etiologies such as infection, systemic diseases, or tumors must be considered, especially in young patients.

Among bone tumors, Ewing′s sarcoma (ES) is the second most frequent malignant neoplasm in children and adolescents, accounting for approximately 10%–15% of all primary bone sarcomas [4, 5]. It typically arises in the diaphyseal region of long bones such as the femur, tibia, and pelvis, while ulna involvement is exceptionally rare [6], which may contribute to diagnostic delays, particularly when early symptoms resemble benign conditions like lateral elbow pain or epicondylitis [7].

This rarity presents a significant surgical challenge, particularly due to the anatomical complexity of the ulna and its critical biomechanical role in maintaining elbow stability and upper limb function [8, 9]. Reconstruction after wide resection of the ulna demands techniques that preserve both mobility and functional integrity while optimizing the patient′s quality of life [10].

First described by Ewing in 1921 [11], this malignancy is characterized by specific chromosomal translocations, most notably t(11;22)(q24;q12), which results in the EWS‐FLI1 fusion gene [12]. This genetic event plays a central role in the tumor′s pathogenesis and is associated with aggressive clinical behavior and a high metastatic potential [13].

Recent clinical reports have emphasized the need for high diagnostic suspicion in young patients presenting with persistent, atypical musculoskeletal pain. Notably, Zanconato et al. reported a case where ES mimicked lateral elbow pain for several months, underscoring the challenge of early recognition in unusual anatomical locations [7].

Several reconstructive strategies have been described for managing ulnar bone defects, including bone grafts (vascularized and nonvascularized), cryopreservation and reimplantation, radial neck transposition, and reimplantation of irradiated autografts [9]. Each technique carries specific benefits and limitations. For example, fibular grafts offer structural stability but may impair forearm rotation [14]. Prosthetic implants can provide good initial outcomes but are often associated with long‐term complications such as infection and implant failure [8]. Bone transposition procedures—such as using the radial neck—have shown functional promise but may be constrained by technical and biomechanical challenges [6, 9].

This article reports a case involving total ulnar resection in a patient with ES, followed by radial head transposition to the olecranon, with reattachment of the triceps tendon [8, 9]. This innovative reconstructive approach aims to preserve elbow functionality and ensure upper limb stability, representing a viable solution in complex oncologic surgical scenarios.

This case report was prepared in accordance with the CARE guidelines for case report publication [14]. Written informed consent was obtained from the patient for the publication of all clinical data and images. Patient privacy and confidentiality were preserved in compliance with the ethical principles outlined in the Declaration of Helsinki [15].

## 2. Surgical Case

A 17‐year‐old male reported intermittent pain in the mid‐forearm and lateral elbow region, with no history of trauma. The pain was mild, activity‐related, and responsive to nonsteroidal anti‐inflammatory drugs (NSAIDs), leading to a presumptive diagnosis of lateral epicondylitis by a primary care physician. Conservative management with rest, analgesics, and physical therapy was maintained for several months.

Approximately 4 months before referral to the specialized center, the clinical picture progressively worsened, presenting with continuous pain, visible swelling, and nocturnal discomfort. At that point, imaging studies performed in the patient′s hometown revealed an osteolytic lesion in the ulna, raising suspicion of a bone tumor.

The patient resides in a rural area of the state of Pará, in the Brazilian Amazon, where there is only one public referral center for orthopedic oncology—located in the capital city, Belém—responsible for serving all 144 municipalities [16] in the state, many of which have territorial dimensions comparable to European countries. This structural limitation imposes significant barriers to early diagnosis and timely access to specialized care. The patient was ultimately referred to the referral hospital after 10 months of symptoms, by which time clinical signs of local disease progression were already evident [17].

The patient′s medical history was unremarkable, with no prior diagnoses or chronic conditions. Given the known genetic mechanisms involved in the pathogenesis of ES—particularly the EWS‐FLI1 translocation—a detailed family history was reviewed. No cases of bone or soft tissue sarcomas, or other oncologic diseases, were reported among first‐ or second‐degree relatives.

On initial physical examination at the orthopedic oncology service, the patient presented with a firm, nonmobile mass in the mid‐third of the right forearm, accompanied by mild tenderness on palpation. There were no signs of neurovascular compromise. Elbow range of motion (ROM) was preserved, as were wrist and hand functions. Distal pulses were intact and symmetrical.

**Figure 1:**
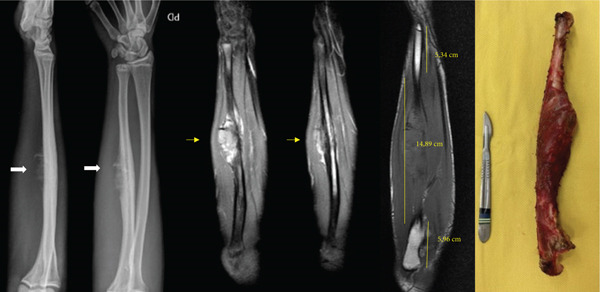
Radiographs, magnetic resonance imaging, and surgical specimen of the right forearm.

Radiographs demonstrated extensive destruction of the ulnar diaphysis, accompanied by a bulky soft tissue component. Magnetic resonance imaging and bone scintigraphy confirmed the presence of an aggressive, locally advanced tumor, with no evidence of distant metastasis. Radiographs revealed an expansile osteolytic lesion in the ulnar diaphysis, prompting initiation of oncologic staging (Figure 1). The surgical staging of the tumor was performed according to the Enneking system, adopted by the Musculoskeletal Tumor Society (MSTS), which considers histological grade, anatomical compartmentalization, and presence of metastasis [18].

The decision to perform a complete ulnar resection, rather than an intercalary reconstruction using either an allograft or a vascularized fibular graft, was based on the tumor′s extent and anatomical limitations for achieving secure bone fixation. On T1‐weighted MRI, the tumor measured approximately 15 cm, extensively involving the ulnar diaphysis. Considering appropriate oncologic margins of 2 cm proximally and distally, less than 5 cm of healthy bone would remain at each end. This challenge was further compounded by the presence of the distal ulnar physis, approximately 1 cm in length, which does not provide adequate mechanical stability for fixation. Attempting to fix a long graft (19 cm) onto such short residual segments would result in a highly unfavorable lever arm, increasing the risk of mechanical failure, nonunion, or fracture. Moreover, using a vascularized fibular graft would require a considerable length, compromising the viability of vascular anastomosis due to the disproportion between the graft and the remaining bone ends. Given these oncologic, anatomical, and biomechanical considerations, complete ulnar resection followed by biological reconstruction using radial neck transposition to the humeral trochlea was selected. This approach proved to be both safe and functionally effective in this clinical context.

**Figure 2:**
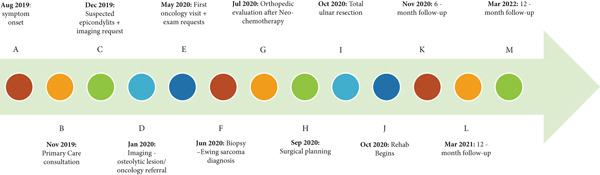
Timeline: (A) August 2019: onset of painful symptoms in the forearm and elbow, with no history of trauma. (B) November 2019: clinical consultation at a primary care facility. (C) December 2019: follow‐up appointment; initial suspicion of epicondylitis and request for imaging exams. (D) January 2020: return visit with elbow imaging, which revealed an osteolytic lesion in the ulnar diaphysis. The patient was referred to oncology through the intermunicipal referral system. (E) May 2020: first oncology consultation, with further diagnostic workup requested (MRI and abdominal and chest CT scan) and ultrasound‐guided needle biopsy. (F) June 2020: ultrasound‐guided soft tissue biopsy identified a “malignant mesenchymal neoplasm composed of small round blue cells, with morphological and immunophenotypic features compatible with Ewing′s sarcoma (ES).” (G) July 2020: orthopedic evaluation with surgical indication following neoadjuvant chemotherapy. (H) September 2020: orthopedic consultation for surgical planning. (I) October 2020: surgery for total ulnar resection. (J) October 2020: initiation of elbow rehabilitation. (K) November 2020: 6‐month follow‐up. (L) March 2021: 12‐month follow‐up. (M) March 2022: 24‐month follow‐up.

After neoadjuvant chemotherapy, a radical surgical approach was selected, involving complete resection of the ulna and functional reconstruction of the forearm using radial neck transposition to the humeral trochlea. The objective was to preserve skeletal architecture and maintain soft tissue tension. The patient and his family were thoroughly informed about the necessity of the procedure and the inherent risks, including partial loss of elbow mobility (Figure 2).

## 3. Surgical Technique

The patient was positioned in the supine position under general anesthesia, with regional nerve block performed at the end of the procedure for postoperative pain control. A pneumatic tourniquet was used after blood exsanguination by limb elevation.

Surgical access was achieved through a direct posterior approach to the ulna, using an elliptical incision that encompassed the previous biopsy tract. Throughout the procedure, the ulnar nerve was carefully identified, dissected, and protected. As the olecranon region was free of tumor, it was possible to resect en bloc the insertion of the triceps tendon along with the posterior joint capsule.

Extensive invasion of the ulnar diaphysis by the tumor was confirmed intraoperatively, making it impossible to obtain wide oncologic margins without complete bone resection. Thus, the ulna was entirely excised, including the affected soft tissues.

For elbow reconstruction, the native articulation between the radius and the humerus was disengaged, allowing posterior mobilization of the radial neck for alignment with the humeral trochlea. During this transposition, we identified the biceps tendon and muscle belly as the anterior anatomical barrier and the triceps tendon as the posterior barrier. Medial and lateral joint stability was restored using the remaining capsular structures, which were anchored to the radial head with transosseous sutures using 5‐0 Ethibond.

It is important to highlight the biomechanical stabilizers in this reconstruction: The biceps acts as the anterior stabilizer, the reconstructed triceps as the posterior stabilizer, and the capsular remnants provide medial and lateral support. Closure was performed in layers with meticulous hemostasis, and a suction drain was placed. ROM was assessed intraoperatively before and after fixation of the radial head to the trochlea (Figure 3).

Figure 3Intraoperative evaluation. (a) Extension, (b) flexion, (c) supination, and (d) pronation.

**Figure:**
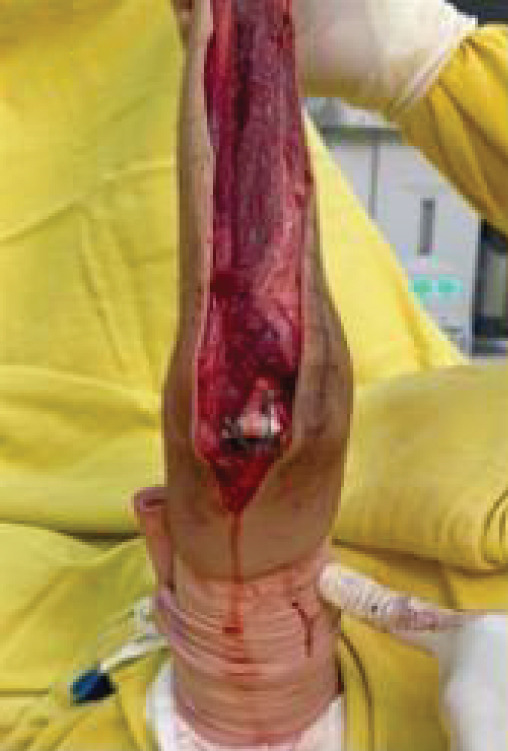
(a)

**Figure:**
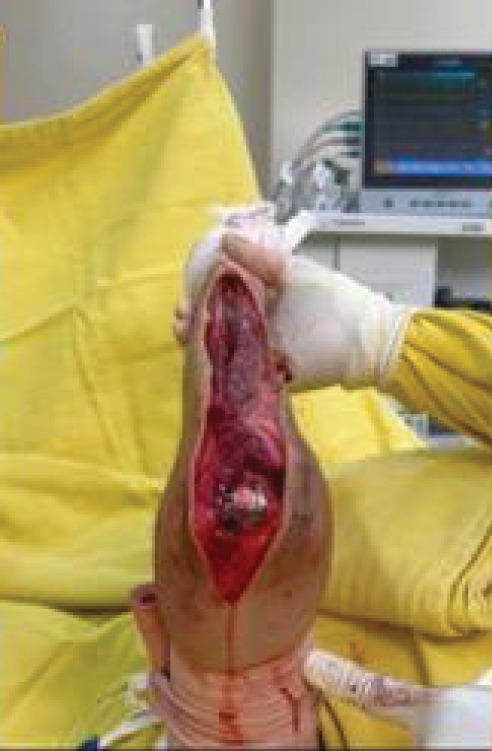
(b)

**Figure:**
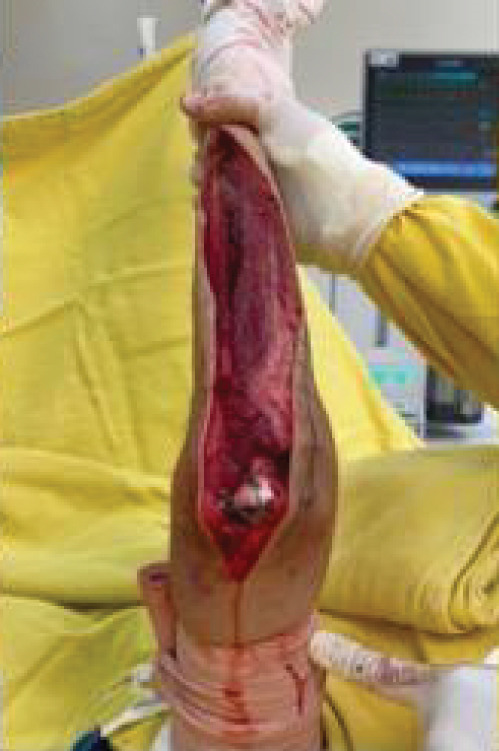
(c)

**Figure:**
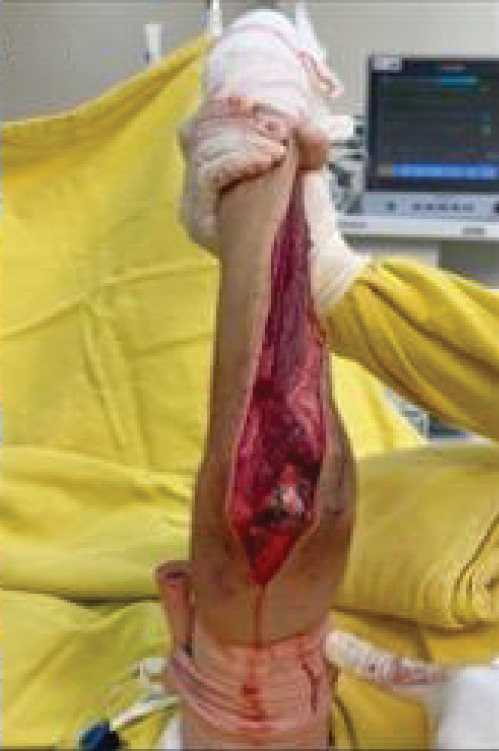
(d)

The patient had an uneventful immediate postoperative course. Outpatient follow‐up was conducted on Postoperative Days 7, 15, and 30. A progressive physiotherapy rehabilitation protocol was initiated, and the patient was referred for continued oncologic follow‐up.

**Figure 4:**
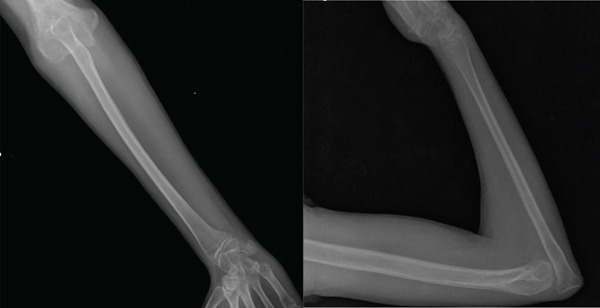
Postoperative radiographs of the right forearm demonstrating complete ulnar resection with preservation of the elbow joint.

Postoperative immobilization was performed using an axillary‐to‐palm splint for 3 weeks, followed by gradual release of assisted movement under physiotherapy supervision. Postoperative radiographs confirmed proper alignment of the operated limb, with stable fixation of the bone transposition (Figure 4).

After 2 years of follow‐up, a comprehensive functional evaluation was conducted using the Enneking classification of the MSTS (Table 1). In addition to the functional parameters assessed, the ROM of the operated limb was measured and compared with the contralateral side, enabling a quantitative analysis of movement arc and any biomechanical restrictions resulting from the surgical procedure. The patient remained free of local recurrence and metastasis, with favorable functional outcomes, absence of significant pain, and preserved hand function (Figure 5 and Table 1).

**Table 1:** Musculoskeletal Tumor Society (MSTS) scoring system for the upper limb.

**Score**	**Pain**	**Functional activity**	**Hand position**	**Dexterity**	**Muscle strength**	**Emotional acceptance**
5	**No pain**	**No restrictions (no disability)**	**Unlimited (can be raised to 180°)**	**No limitations**	Normal load (muscle strength Grade 5)	**Enthusiastic**
3	Moderate/no disabling	Recreational restriction (mild disability)	Cannot elevate above shoulder or lacks pronation/supination (up to 90°)	Loss of fine movements	**Light load (muscle strength Grade 3)**	Satisfied
1	Moderate/intermittently disabling	Partial occupational restriction (severe disability)	Cannot elevate above waist (up to 30°)	Unable to perform pinching	Cannot overcome gravity (strength Grade 2)	Accepts the condition
0	Severe/continuously disabling	Total occupational restriction (complete disability)	None (elevation limited to 0°)	Unable to grasp	No movement (muscle strengths 0–1)	Dissatisfied

*Note:* Bold text indicates the patient′s reported symptoms and the corresponding category selected in the MSTS (Musculoskeletal Tumor Society) scoring system.

Figure 5Postoperative evaluation during outpatient follow‐up after 2 years. (a) Extension, (b) flexion, (c) supination, and (d) pronation.

**Figure:**
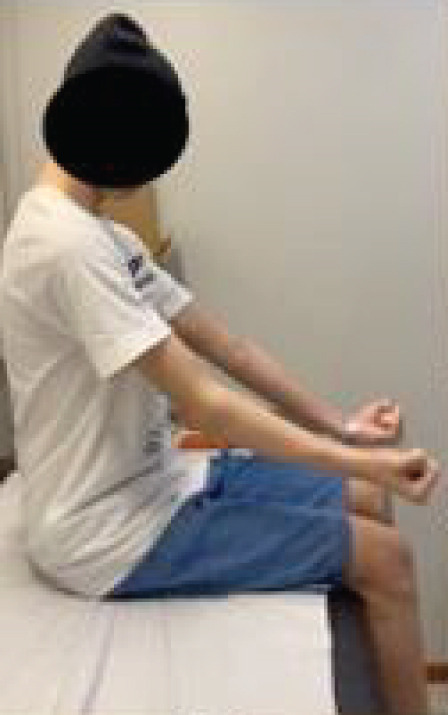
(a)

**Figure:**
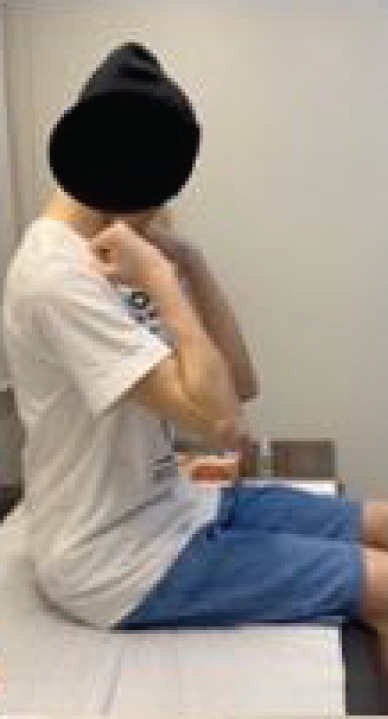
(b)

**Figure:**
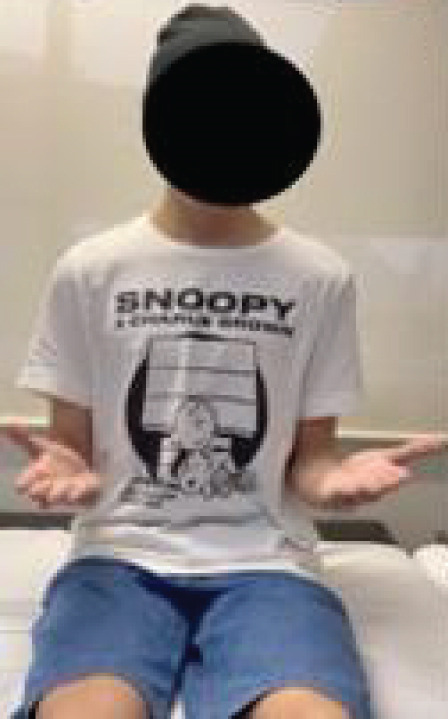
(c)

**Figure:**
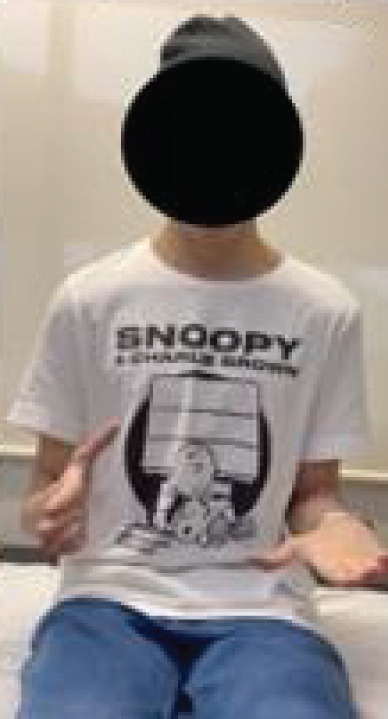
(d)

Outcome assessment performed 2 years after surgery during outpatient follow‐up. The patient reported absence of pain, no recreational limitations, and was able to elevate the hand to 180° in the frontal plane without restrictions in pronation or supination. Fine motor skills, pinch, and grip were preserved, with muscle strength graded as 5. The patient expressed enthusiasm regarding the outcomes and achieved a total of 28 out of 30 points on the MSTS scoring system, as highlighted in bold.

## 4. Discussion

Lateral elbow pain is a common complaint in orthopedic practice, typically attributed to benign conditions such as epicondylitis or overuse injuries. However, in rare instances, it may represent the initial manifestation of a serious underlying pathology, including primary bone malignancies such as ES.

Ulnar reconstruction following oncologic resection poses a considerable surgical challenge, particularly when the goal is to preserve elbow stability and upper limb function. This report presents a case of complete ulnar resection in a 17‐year‐old patient diagnosed with ES. A limb‐salvage procedure was performed using radial neck transposition to the humeral trochlea—a technique previously described in complex cases of oncologic resection and severe trauma [4].

The literature on primary bone tumors of the ulna is scarce, as they account for only 1% of bone neoplasms [5]. Data from the Rizzoli Institute indicate that, among 19,689 cases of primary bone tumors, ulnar involvement occurred in only 0.9% [6]. Similarly, Joo et al. described 24 cases of primary ulnar bone tumors in a retrospective study [13]. Our report highlights the importance of exploring innovative reconstructive strategies for treating rare ulnar bone tumors, particularly in scenarios where amputation is conventionally indicated.

Given the lack of standardized guidelines for managing these lesions, the technique described represents a promising alternative that allows for limb preservation and functional restoration of the elbow. By offering a viable solution for complex cases, our study contributes to expanding therapeutic options, minimizing morbidity associated with extensive resections, and broadening the prospects for functional rehabilitation. Additionally, it offers advantages in being simple, cost‐effective, time‐efficient, and highly applicable.

The ulna has a thick cortical structure along the diaphysis, which provides greater mechanical strength, while its proximal portion features thinner cortical layers and a transition to trabecular bone. These structural characteristics influence tumor growth patterns, as diaphyseal lesions are more likely to be detected early due to intense pain [8], whereas tumors in the proximal region may reach larger volumes before diagnosis. In a previous case series, 85.7% of diaphyseal tumors were initially identified due to pain, while 27.3% of cases involved the proximal ulna [9]. These findings reinforce the importance of clinical and imaging evaluation for early detection and surgical planning.

Radial neck transposition has been described as an effective alternative to preserve elbow function and avoid complications associated with other reconstruction methods, such as bone grafts and prosthetic implants [10]. Previous studies have shown that this technique can result in a functional range of motion between 35° and 135°, with maintained joint stability and no chronic pain [9]. However, a reduction in flexion and extension strength has been observed, often limited to 50% of the contralateral limb′s strength, due to the absence of a suitable insertion point for the flexor muscles [19].

The decision between radial neck transposition and other reconstructive techniques, such as vascularized fibular grafts, should consider factors like long‐term durability and risk of complications. Puri et al. [20] reported that patients who underwent biological reconstructions achieved a functional arc of 10° to 130° after 5 years of follow‐up, whereas patients treated with prosthetic implants had an average arc of 30°–110° [21, 22]. Similarly, Gulia et al. [8] demonstrated that patients reconstructed with prostheses had higher rates of mechanical complications, infection, and implant failure.

Another critical factor is the impact of postoperative immobilization. Aljuhani et al. observed that patients whose elbows were immobilized at 90° for 3 months showed poorer functional recovery [23]. In contrast, studies that implemented early mobilization protocols reported better outcomes, with a lower incidence of joint stiffness [24, 25].

Elbow prostheses are frequently used in the reconstruction of periarticular tumors. However, they are associated with significant complication rates. Gulia et al. analyzed 19 patients undergoing elbow prosthetic reconstruction and reported reductions in pain and improved mobility (flexion–extension range from 30° to 80°); nevertheless, 22.2% of patients experienced failure of the reconstruction [8]. The most common complications include infection, implant loosening, and periprosthetic fractures, which often require revision surgeries and negatively impact functional prognosis [26, 27].

Moreover, prosthetic reconstruction requires adequate soft tissue coverage, which can be a limiting factor in extensive resections such as the one presented in this study [28]. Given these challenges, we opted for a biological technique, which offers better long‐term functional preservation rates.

One of the main limitations of this report is the absence of long‐term follow‐up data, a critical factor in assessing the overall success of the reconstruction. Currently, the literature on long‐term functional outcomes of radial neck transposition is scarce, underscoring the need for extended follow‐up to evaluate the durability of the reconstruction and its impact on quality of life [29].

Functional data available in the literature suggest that patients undergoing this technique may experience progressive recovery, although outcomes vary depending on the duration of immobilization, fixation method, and the integrity of the surrounding musculature [29]. Therefore, continued monitoring of this patient will provide greater insight into the biomechanical and functional outcomes of the technique and may support decision‐making in similar clinical scenarios.

Limb preservation through biological reconstructions, such as radial neck transposition to the humeral trochlea, represents a promising option with favorable functional outcomes and a lower risk of complications compared to prostheses and bone grafts. However, prospective studies with long‐term follow‐up remain essential to determine the true biomechanical and clinical impact of the technique.

Functional assessment performed 2 years after surgery demonstrated an excellent clinical and functional outcome for the patient who underwent complete ulnar resection and elbow reconstruction using radial neck transposition. With a score of 28 out of 30 on the MSTS scale, the patient achieved near‐complete recovery of upper limb function, confirming the effectiveness of the chosen technique.

The absence of pain and the ability to elevate the hand to 180° in the frontal plane, without significant restrictions, indicate that radial neck transposition successfully preserved joint mobility and biomechanical elbow stability. However, a specific limitation in supination was observed, with the range reduced by approximately half, while pronation remained preserved. This restriction may be attributed to the absence of the ulna, which normally serves as a stabilizing axis for pronation–supination movements, and to the altered biomechanics of the reconstructed radiohumeral joint.

Previous studies on similar biological reconstructions report that pronation–supination is often the most affected movement due to altered dynamics between the radius and ulna following extensive resections [3–5]. Although limited supination may have a functional impact on tasks requiring this motion—such as holding objects in the anatomical position or turning keys—the overall functionality of the limb was preserved, including grip strength, pinch, and fine motor skills. These findings are consistent with the literature, which emphasizes that well‐planned biological reconstructions tend to yield better functional outcomes compared to prosthetic solutions, which are frequently associated with higher rates of mechanical complications and infections [8, 13, 20, 27].

In addition to biomechanical aspects, the patient′s positive emotional acceptance—classified as “enthusiastic”—highlights the favorable impact of the technique on quality of life. Limb preservation, particularly with good mobility and hand functionality, is associated with better psychological adjustment compared to amputation or less satisfactory reconstructions [9, 10].

Despite the promising results, some questions remain. The limitation in supination may affect more specific tasks, and the absence of long‐term follow‐up prevents a definitive assessment of the reconstruction′s durability. Additional studies using dynamometry and more detailed biomechanical testing would help quantify muscle strength and functional compensation in the operated forearm.

## 5. Conclusion

The findings of this study reinforce that radial neck transposition represents a viable and functionally effective alternative for elbow reconstruction following oncologic resection of the ulna, offering preserved mobility with minimal functional limitation. Despite some restriction in supination, the patient maintains adequate muscle strength, preserved grip function, and independence in daily activities. These results suggest that the technique may be a promising option for limb preservation in cases involving wide ulnar resection. However, further studies and long‐term follow‐up are essential to assess the functional stability and durability of the reconstruction over time.

## Ethics Statement

This study was conducted in accordance with the Declaration of Helsinki. The study protocol received institutional ethics committee approval. Written informed consent was obtained from the patient and/or legal guardian.

## Consent

Written informed consent for publication was obtained from the patient and/or legal guardian.

## Disclosure

The study was conducted at Octávio Lobo Pediatric Oncology Hospital, Belém, Pará, Brazil.

## Conflicts of Interest

The authors declare no conflicts of interest.

## Author Contributions

F.B.C.F. and D.R.S. were responsible for the surgical design of the case, clinical follow‐up of the patient, and critical review of the manuscript. L.C.C.B. participated in the surgical procedure, provided technical supervision, and critically reviewed the intellectual content. F.G.M. contributed to the reconstructive planning and comparative analysis with the literature. H.R.R.N. conducted the literature review, organized the clinical images, and contributed to data organization. R.D.S.C. assisted in the technical planning of the case and contributed to the biomechanical discussion. D.R.S. additionally coordinated the project and was responsible for the full writing and scientific structuring of the manuscript, as well as correspondence with the journal. L.G.C. contributed to the literature review and critical analysis of the functional outcome. E.G.N. and R.M.B. were responsible for outpatient follow‐up, clinical data collection, and methodological support.

## Funding

No funding was received for this manuscript.

## Data Availability

The data that support the findings of this study are available on request from the corresponding author. The data are not publicly available due to privacy or ethical restrictions.
